# Genetic diversity of lactase persistence in East African populations

**DOI:** 10.1186/s13104-015-1833-1

**Published:** 2016-01-04

**Authors:** Hisham Y. Hassan, Anke van Erp, Martin Jaeger, Hanan Tahir, Marije Oosting, Leo A. B. Joosten, Mihai G. Netea

**Affiliations:** Banoon ART and Cytogenetics Centre, Bahrain Defence Force Hospital, Manama, Kingdom of Bahrain; Department of Internal Medicine and Radboudumc Center for Infectious Diseases, Radboud University Medical Center, Nijmegen, The Netherlands; Sudan Medical and Scientific Research Institute, University of Medical Sciences and Technology, Khartoum, Sudan; Department of Internal Medicine (463), Radboud University Medical Center, P.O. Box 9101, 6500 HB Nijmegen, The Netherlands

**Keywords:** Lactase, East Africa, Sudan, Nilotes

## Abstract

**Background:**

The expression of lactase which digests lactose from milk in humans is generally lost after weaning, but selected mutations influencing the promoter of the lactase gene have spread into the human populations. This is considered a classical example of gene-culture co-evolution, and several studies suggested that the lactase gene has been under strong directional evolutionary selective pressure in the past 5000 to 10,000 years.

**Results:**

In the present study we investigated the distribution of three gene variants leading to lactase persistence in 12 different East African populations as well as one European population. Our results show that with the exception of Copts and Nilotic populations who are fully lactose non-persistent, the majority of populations of East Africa show at least partly lactose persistence, with both ethnic and socio-economic aspects playing an important role in the distribution of genetic variants. In this study, the variants C/G-13907 and T/G-13915, which are the major variants among the nomadic Arabs in the Arabia and Beja of East Africa, showed remarkable frequencies in Sudanese populations, especially those of pastoralists, in line with the historical links and bidirectional migration of nomadic populations between Arabia and East Africa. The C/T-13910 variant, generally associated with European populations is uniquely present among the Fulani.

**Conclusions:**

These data indicate that a combination of socio-economic, ethnic and evolutionary factors converged to shape the genetic structure of lactase persistence in East African populations.

**Electronic supplementary material:**

The online version of this article (doi:10.1186/s13104-015-1833-1) contains supplementary material, which is available to authorized users.

## Background

The milk sugar lactose requires hydrolysis in the intestinal tract into the monosaccharides glucose and galactose in order to facilitate their absorption by the enterocytes. The breakdown of lactose in the small intestine is catalyzed by the enzyme lactase, a glycoside hydrolase, which is encoded in humans by the *LCT* gene [1]. Lactase persistence represents the continued ability to break down lactose in adults, and is a dominant Mendelian polymorphic trait identifiable through its phenotypic effect lactose tolerance. Globally, the percentage of lactase persistence haplotype is approximately 35 %, with the highest frequency being found among European and Americans of European origin and its lowest frequencies present in sub-Saharan Africa and southeast Asia [2–4].

The ability of adult humans to digest lactose sugar and the nutritional benefit conferred by this trait (by using milk as an additional energy source) is considered as a classical example of gene-culture co-evolution [5, 6]. Many studies suggested that the *LCT* gene has been under strong directional evolutionary selective pressure resulting from shared cultural traits, animal domestication and adult milk consumption within the past 5000–10,000 years [7–10]. The phenotype of lactase persistence is due to the presence of a haplotype extending more than 2 M nucleotide base pairs on chromosome 2. To date, five major variants (C/T-13910, C/G-13907, T/G-13915, T/G-14009 and G/C-14010) that originated on different haplotype backgrounds in intron 13 of the *MCM6* gene are known to regulate the *LCT* enhancer and are associated with lactase persistence in humans: the C/T-13910 SNP found mainly in Europeans, and C/G-13907, T/G-13915 and G/C-14010 SNPs found in African and Middle Eastern populations, while T/G-14009 is found in Ethiopia [2, 10–13]. The association of these genotypes with the phenotype of lactose tolerance has recently been confirmed for African populations [13, 14].

The link between domestication of cattle and the inhabitants of the Nile Valley has a long history [15]. Archeological excavations revealed that people of Neolithic and Kerma who lived in the present day northern Sudan and southern Egypt some 5000 years ago raised livestock from which around 80 % were cattle [16, 17]. Moreover, historical and archeological findings show evidence of milk fermentation by the later communities as well. Interestingly, drawings attributed to people of the ancient Kingdom of Meroe (690 BC to AD 323) are dominated by cattle. Also the classical Greek writer Strabo (7 BC) mentioned that the Meroites, which are called Ethiopians by classical writers, used fermented milk in their foods. This information indicates that cattle were the major livestock of ancient kingdoms of the Nile Valley [18, 19]. Today, consumption of fresh and fermented milk is widespread in East African populations, including pastoral and agriculturalist communities in the Sudan, South Sudan and Ethiopia. Milk and its products play an important role in the traditional diet of the people, and serve as a major source of cash income for most families. Cattle are the primary source of milk in the region, although nomadic populations also raise sheep, goats and camels [17, 20].

The aim of the present study was to assess the presence of three *MCM6* variants modulating *LCT*. Variation in these SNPs lead to the tolerance to lactose in different populations from East Africa living along the Nile Valley in the current territory of the Sudan, South Sudan and Ethiopia. We correlated this information with the occupational characteristics of each of the populations, as well as with ethnic and historical aspects, to increase our understanding of population history in this ancient space of human civilizations.

## Methods

To investigate the genetic diversity of lactase persistence alleles among East African populations, a total of 488 saliva samples of unrelated individuals from 12 different East-African ethnic groups from the Sudan, South Sudan and Ethiopia were collected from different geographical area in the Sudan prior to separation.

The samples used in the present study were collected and studied with ethical approval and informed consent (ref. SUM/2010/7). All protocols were approved by the IRB of the Sudan Medical and Scientific Research Institute (SUMASRI) at the University of Medical Sciences and Technology in Khartoum, and were carried out in accordance with the approved guidelines.

Appropriate informed consent and questionnaires were obtained from all participants before sample collection; and samples were grouped according to their self-declared ethnic background of parents and grandparents, and languages. Sample sizes, geographic origin, and linguistic affiliation for each population are reported in Table 1. Saliva samples were collected using OrageneTM collection kits (DNA Genotek, Ontario, Canada). The DNA isolation, purification, and genotyping were performed at Radboud University Medical Center, Nijmegen, The Netherlands, following the manufacturer’s standard protocols.

**Table 1:** Geographic locations, sample size, socio-economic activities and linguistic affiliation of populations analyzed for lactase haplotypes in this study

Populations	Sub-group	Country	Sample size	Socio-economic activities	Linguistic affiliation	Sub-family	Location	Coordinates
Arabs	Gaalien	Sudan	40	Agriculturalists	Afro-Asiatic	Semitic	Shendi	16N 33E
	Shwaiga	Sudan	38	Agriculturalists	Afro-Asiatic	Semitic	Karima	18N 31E
	Shokrya	Sudan	40	Pastoralists	Afro-Asiatic	Semitic	New Halfa	15N 35E
Beja	Bani Amir	Sudan	37	Pastoralists	Afro-Asiatic	Cushitic	Sinkat	18N 36E
Copts	–	Sudan	39	Agriculturalists	Afro-Asiatic	Ancient Egyptian	Khartoum	15N 32E
Nubians	Halfawien	Sudan	39	Agriculturalists	Nilo-Saharan	Eastern Sudanic	Wadi Halfa	21N 31E
	Mahas	Sudan	39	Agriculturalists	Nilo-Saharan	Eastern Sudanic	Kerma	19N 30E
Darfurians	Mixed	Sudan	49	Agriculturalists	Nilo-Saharan	Fur and Eastern Sudanic	El-Fashir	13N 25E
Nuba	Mixed	Sudan	40	Agro-pastoralists	Nilo-Saharan and Niger-Congo	Eastern Sudanic, Kordofanian and Kadugli-Krongo	Kadugli	11N 29E
Fulani	–	Sudan	39	Pastoralists	Niger-Congo	Atlantic	Nomadic groups	–
Nilotes	Mixed	South Sudan	48	Agro-Pastoralists	Nilo-Saharan	Eastern Sudanic	Juba	4N 31E
Ethiopians	Amhara, Tigray and Oromo	Ethiopia	40	Agro-Pastoralists	Afro-Asiatic	Semitic and Cushitic	Khartoum	15N 32E
Dutch	–	Netherlands	46	Agro-Pastoralists	Indo-European	Germanic	Dutch groups	–

All samples were genotyped for three SNPs in the MCM6 gene, which have previously been shown to regulate transcriptional activity of the LCT promoter and therefore confer tolerance to lactose (C/G-13907 (rs41525747), C/T-13910 (rs4988235) and T/G-13915 (rs41380347)) [10]. SNPs were genotyped using Sanger sequencing, and for C/T-13910 we additionally performed TaqMan genotyping to confirm the Sanger sequencing results. In order to compare the African samples with samples from European origin, we genotyped the MCM6 SNPs in DNA isolated from blood samples collected from 46 healthy Dutch volunteers. Isolation was performed using the Gentra Pure Gene Blood kit (Qiagen, Venlo, The Netherlands) following the manufacturer’s protocol. Data analysis was performed using Arlequin v.3 and Genetic Data Analysis v.1.0 programs [21, 22]. The data set supporting the results of this article summarized in the Additional file 1 of this article.

## Results

**Fig. 1:**
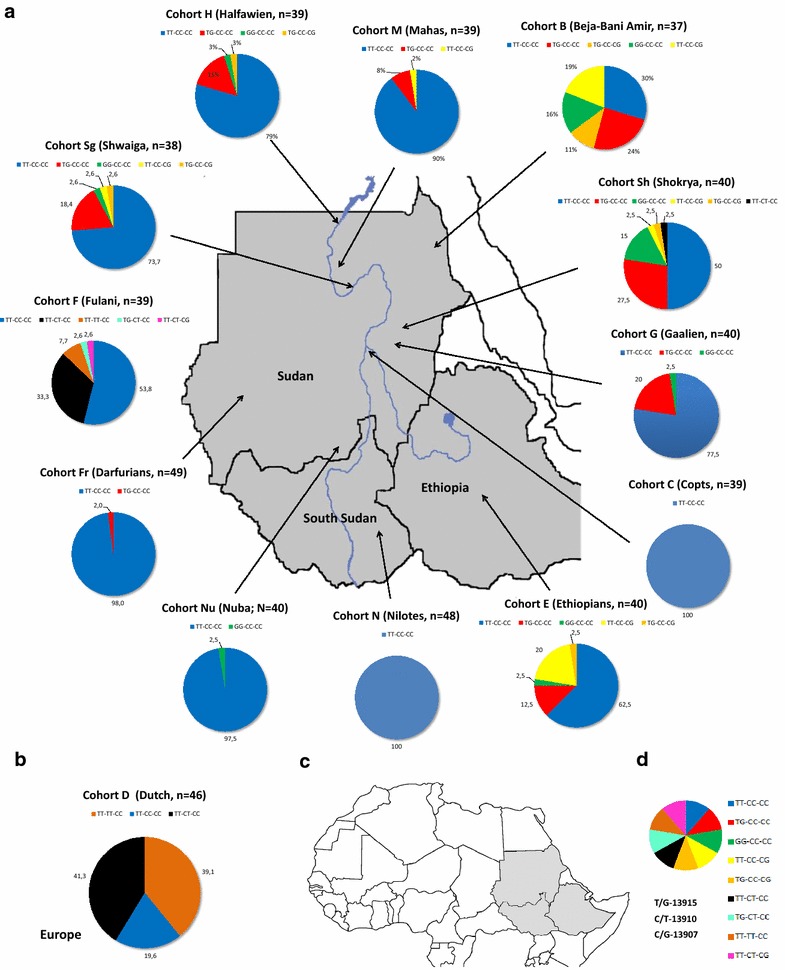
Distribution of MCM6 haplotypes among different East African populations: **a** Pie charts representing the proportion of each haplotype among the selected populations in this study. The* arrows* show the approximate locations of the populations. The Fulani is a nomadic group and has no specific geographical location. **b** We additionally added a European (Dutch) cohort to compare the different groups. **c** The location of the Sudan, South Sudan and Ethiopia and the neighboring countries in Africa. **d** The key and distribution of LP haplotypes, columns of bases represent the genotypes of T/G-13915, C/T-13910 and C/G-13907 respectively. Modified from d-maps.com

The genotype and haplotype frequencies of the SNPs conferring lactase persistence among the selected populations are shown in Fig. 1 and Table 2 as well as Additional file 1: Table S1. The variants T/G-13915, C/G-13907 and C/T-13910 are found among East African populations with frequencies of 15.5, 5.5 and 4 % respectively. The C/T-13910 allele, which is the most common in European populations, was identified in high frequency among Dutch (80 %), Fulani (46 %) and Shokrya (2.5 %). The variants C/G-13907 and T/G-13915, which were found previously in Arabia and among Beja of East Africa [1, 10–12, 23]. were found in high frequencies among Beja, Arabs, Nubians and Ethiopians. None of the three genotyped variants was observed in Copts of the Sudan or Nilotes of South Sudan.

**Table 2:**
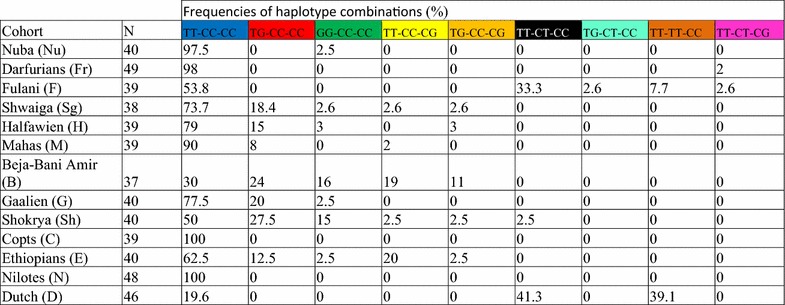
Frequencies of haplotype combinations of the three LP variants, T/G-13915, C/T-13910 and C/G-13907 respectively among the populations genotyped in this study

The SNPs in the various populations were tested for Hardy–Weinberg Equilibrium and pairwise disequilibrium using the three variants C/G-13907, C/T-13910 and T/G-13915. The Copts and Nilotes were excluded from the analysis due to the absence of any variants among the genotyped population. The values of observed and expected heterozygosity and fixation indices and their significance are shown in Table 3. No locus of the three polymorphic loci showed a significant departure from HWE with one exception of the variant T/G-13915 in Nuba (*P* = 0.01). In addition, there was no significant pairwise disequilibrium between loci, with the only exception found between C/T-13910 and T/G-13915 (*P* = 0.01), and between C/G-13907 and T/G-13915 (*P* = 0.01) in Nuba.

**Table 3:** Frequencies of expected and observed heterozygosity and fixation index for the LP variants C/G-13907, C/T-13910 and T/G-13915 among ten ethnic groups of East Africa and Dutch from Europe

Populations (N)	C/G-13907			C/T-13910			T/G-13915		
	He	Ho	F (P)	He	Ho	F (P)	He	Ho	F (P)
Gaalien (40)	0.000	0.000	–	0.000	0.000	–	0.202	0.225	−0.114 (>0.05)
Shwaiga (38)	0.052	0.053	−0.014 (>0.05)	0.000	0.000	–	0.232	0.211	0.092 (>0.05)
Shokrya (40)	0.049	0.050	−0.013 (>0.05)	0.025	0.025	0.000 (>0.05)	0.425	0.300	0.297 (>0.05)
Beja-Bani Amir (37)	0.257	0.297	−0.161 (>0.05)	0.000	0.000	–	0.454	0.351	0.228 (>0.05)
Halfawien (39)	0.026	0.026	0.000 (>0.05)	0.000	0.000	–	0.186	0.205	−0.101 (>0.05)
Mahas (39)	0.026	0.026	0.000 (>0.05)	0.000	0.000	–	0.075	0.077	−0.027 (>0.05)
Darfurians (49)	0.000	0.000	–	0.000	0.000	–	0.020	0.020	0.000 (>0.05)
Nuba (40)	0.000	0.000	–	0.000	0.000	–	0.049	0.000	1.000 (=0.01)
Fulani (39)	0.026	0.026	0.000 (>0.05)	0.398	0.385	0.036 (>0.05)	0.026	0.026	0.000 (>0.05)
Ethiopians (40)	0.202	0.225	−0.114 (>0.05)	0.000	0.000	–	0.182	0.150	0.179 (>0.05)
Dutch (46)	0.000	0.000	–	0.486	0.413	0.152 (>0.05)	0.000	0.000	–

*N* sample size, *He* expected heterozygosity, *Ho* observed heterozygosity, *F* fixation index

## Discussion

In the present study variable frequencies in the distribution of *MCM6* genetic variants that influence expression of *LCT* gene were identified in selected East African ethnic groups living in the present territory of the Sudan, South Sudan and Ethiopia (see Table 1). Shokrya, Fulani and Beja-Bani Amir of the Sudan show the highest genetic diversity of LP haplotypes. These three groups are pastoralist, and milk consumption is an important part of their diet. We can thus assume that genotyping data support the hypothesis of a significant selective advantage of lactose absorption. Interestingly, the European haplotype C/T-13910 is uniquely present in considerable frequencies among the Fulani; which may suggest the possible gene flow between Fulani and European populations, or a relatively recent back migration of this group to Africa [24–26]. Remarkably, a notable frequency of the “T” allele was shown in the Arab nomadic group Shokrya, which may be a sign of gene flow from Fulani into this group as a result of their wide distribution in the West and East of the Sudan, which is part of Africa’s Sahel.

Both Nilotes and Copts show the absence of lactase persistence alleles. Nilotes are inhabited in the South Sudan and are agro-pastoralists who dwell in the savannas region. Nilotes as well as Nuba breed cattle, but animals are kept not so much for the production of meat and milk, but rather as symbols of wealth and social standing (cows are used for paying marriage dowry) [28].

Nilotes are of unusually large stature, and live in the savannas of the Upper Nile region. They comprise Dinka, Nuer, Shilluk, Anyuak, Baria and a number of small ethnic groups. Nilotes speak languages within the Nilo-Saharan family. Farming and hunting are the major nutritional sources in Dinka and Nuer, populations that breed cattle and lead a semi-nomadic life, while Shilluk, Baria and Anyuak are agriculturalists [27, 28].

The Copts (ancient Egyptians) descend from longstanding cultures of city-states and empires along the Nile, the socio-activities of this group being farming and trade. These different socio-cultural aspects of Copts, Nilotes and Nuba, most likely combined with the serendipitous lack of arising of novel *LCT* mutations, explain the absence/low frequencies of LP alleles among these ancient sedentary populations of the Nile Valley.

The other residential groups of the Nile (Shwaiga, Gaalien, Mahas and Halfawien) are agriculturists. Despite the fact that their economy is mainly agricultural, they exhibit moderate allele frequencies of LP. This is intriguing in the sense that one would expect sedentary populations to be more able to admix and receive genes from their nomadic counterparts, as the area surrounding the Nile is economically attractive. This assumption is supported by the negative values of the fixation indices of these groups, which may indicate the increase of heterozygosity. Arabs, Nubians and Beja of the Sudan, and Ethiopians show high frequencies of the allele T/G-13915, which is the major LP variant among the nomadic Arabs in Arabian Peninsula [23]. This supports the historical link and bidirectional migration of nomadic populations between Arabia and East Africa. And the significant frequencies of this allele of the nomadic Arabs among Nubians of the Sudan may be due to the gene flow form Afro-Asiatic into this Nilo-Saharan group. Nubians reside at the entering port of the Sudan along the Nile at the border with Egypt, where they were influenced by Arabs as a direct result of the penetration of large numbers of Arabs from Arabia and Egypt into the Sudan over a long period of time following the arrival of Islam around 651 AD [29].

Ethiopians genotyped in this study consist of three major groups, Oromo, Amhara and Tigray, they practice farming and animal breeding. Ethiopians display a genetic similarity with Beja of the Sudan; both of them have significant frequencies of lactase persistence haplotypes.

Darfurians is a collective term for a number of residential agriculturalist groups living in arid areas in west of the Sudan (Darfur). Most of the groups in the area practice seasonal cultivation of crops during rainy seasons. They do not rely on fresh milk which is likely the reason why they have low proportion of lactase persistence alleles.

Fresh milk is commonly used in pastoralist communities, and although agriculturalists of the Sudan, South Sudan and Ethiopia show lower frequencies of lactase persistence compared to pastoralists, the milk products are widely consumed by all socio-economic classes of East African agriculturalists. Both pastoralists and non-pastoralists exhibit different cultures of fermented milk products, including *Rob* (sour yogurt) in the Sudan and South Sudan, white cheese, *Mish* and *Gariss* in the Sudan, and *Ergo* in Ethiopia. *Rob* and *Ergo* are naturally fermented cow’s or goat’s milk products [20, 30, 31]. They are the most common traditional dairy fermented food in many areas in the Sudan and Ethiopia. The bulk of these products are produced by the nomadic and semi-nomadic communities during the rainy seasons when fresh milk is available in abundance. The milk is fermented using traditional procedures to obtain sour milk and butter. The white cheese and *Mish* are known in urban communities, and they were probably introduced into the Sudan from Egypt during the Turkish rule in the 19th Century. The major forms of milk fermentation give a good example of the integration of Arab and African cultures in the Sudan [17]. *Gariss* is the special fermented milk of Arab camel herders in east and west of the Sudan. The camel herders spend sometimes months sustained on *Gariss* alone [32]. Acetic acid bacteria and yeasts are involved in the fermentation of fresh milk. Starter culture of fermented milk contains lactose and phosphorylation enzymes produced by lactic acid bacteria to hydrolyze lactose and to initiate the lactic acid glucose phosphate energy cycles. Lactose is hydrolyzed to the monosaccharides, glucose and galactose. The galactose is converted in the milk to glucose, which may facilitate the absorption of it in the small intestine among the lactose intolerant people with no known symptoms of malabsorption of milk [32].

In conclusion, in the present study we have investigated the distribution of *LCT* gene variants that are associated with LP in twelve different East African populations. With the exception of Copts and Nilotes who are fully lactose intolerant, the majority of populations of East Africa have lactase persistence at moderate frequency. The genetic cause of lactose tolerance is however variable, with both ethnic and socio-economic aspects playing an important role in the distribution of genetic variants. The allele T/G 13915, the major LP variant among the nomadic Arabs in Arabian Peninsula, shows high frequencies in Sudanese populations, in line with the historical links and bidirectional migration of nomadic populations between Arabia and East Africa.

## Conclusion

These data tally with the previous data of the distribution of lactase persistence in East Africa, although future studies are warranted to investigate the distribution of more LP variants among the populations studied here. In conclusion, our study indicates that a combination of life style/culture with evolutionary pressure played a crucial role in shaping genetic structure of LP in African populations.

## Additional file

10.1186/s13104-015-1833-1 Genomic Data.

## Abbreviations

LP: lactase persistence
SNP: single nucleotide polymorphism

## References

[1] Swallow D, Hollox EJ (2000). The genetic polymorphism of intestinal lactase activity in adult humans. 8th ed. The metabolic and molecular basis of inherited disease.

[2] Bersaglieri T, Sabeti PC, Patterson N, Vanderploeg T, Schaffner SF, Drake JA (2004). Genetic signatures of strong recent positive selection at the lactase gene. Am J Hum Genet.

[3] Ingram CJ, Mulcare CA, Itan Y, Thomas MG, Swallow DM (2009). Lactose digestion and the evolutionary genetics of lactase persistence. Hum Genet.

[4] Itan Y, Jones BL, Ingram CJ, Swallow DM, Thomas MG (2010). A worldwide correlation of lactase persistence phenotype and genotypes. BMC Evol Biol.

[5] Cavalli-Sforza, Luigi L, Menozzi P, Piazza A (1994). The history and geography of human genes.

[6] Hollox E (2005). Evolutionary genetics: genetics of lactase persistence–fresh lessons in the history of milk drinking. Eur J Hum Genet.

[7] Hollox EJ, Poulter M, Zvarik M, Ferak V, Krause A, Jenkins T (2001). Lactase haplotype diversity in the Old World. Am J Hum Genet.

[8] Enattah NS, Sahi T, Savilahti E, Terwilliger JD, Peltonen L, Jarvela I (2002). Identification of a variant associated with adult-type hypolactasia. Nat Genet.

[9] Poulter M, Hollox E, Harvey CB, Mulcare C, Peuhkuri K, Kajander K (2003). The causal element for the lactase persistence/non-persistence polymorphism is located in a 1 Mb region of linkage disequilibrium in Europeans. Ann Hum Genet.

[10] Tishkoff SA, Reed FA, Ranciaro A, Voight BF, Babbitt CC, Silverman JS (2007). Convergent adaptation of human lactase persistence in Africa and Europe. Nat Genet.

[11] Imtiaz F, Savilahti E, Sarnesto A, Trabzuni D, Al-Kahtani K, Kagevi I (2007). The T/G 13915 variant upstream of the lactase gene (LCT) is the founder allele of lactase persistence in an urban Saudi population. J Med Genet.

[12] Ingram CJ, Elamin MF, Mulcare CA, Weale ME, Tarekegn A, Raga TO (2007). A novel polymorphism associated with lactose tolerance in Africa: multiple causes for lactase persistence?. Hum Genet.

[13] Ranciaro A, Campbell MC, Hirbo JB, Ko WY, Froment A, Anagnostou P (2014). Genetic origins of lactase persistence and the spread of pastoralism in Africa. Am J Hum Genet.

[14] Jones BL, Oljira T, Liebert A, Zmarz P, Montalva N, Tarekeyn A (2015). Diversity of lactase persistence in African milk drinkers. Hum Genet.

[15] Macdonald KC (2000). The origins and development of African livestock, Archaeology, Genetics, Linguistics and Ethnography.

[16] RH (1987). Socioeconomic differentiation in the Neolithic Sudan. Cambridge monographs in African archeology 20.

[17] Abdelgadir WS, Ahmed TK, Dirar HA (1998). The traditional fermented milk products of the Sudan. Int J Food Microbiol.

[18] Krzyżaniak L, Kobusiewicz M (1984). The fauna of the Neolithic site of Kadero (Central Sudan). Origin and early development of food-producing cultures in north-east Africa.

[19] Collins RO, Burns JM (2013). A history of Sub-Saharan Africa.

[20] Gonfa A, Foster HA, Holzapfel WH (2001). Field survey and literature review on traditional fermented milk products of Ethiopia. Int J Food Microbiol.

[21] Excoffier L, Laval G, Schneider S (2005). Arlequin (version 3.0): an integrated software package for population genetics data analysis. Evol bioinforma.

[22] Lewis PO, Zaykin D. Genetic data analysis: computer program for the analysis of allelic data. 2001.

[23] Al-Abri AR, Al-Rawas O, Al-Yahyaee S, Al-Habori M, Al-Zubairi AS, Bayoumi R (2012). Distribution of the lactase persistence-associated variant alleles -13910* T and-13915* G among the people of Oman and Yemen. Hum Biol.

[24] Cruciani F, Santolamazza P, Shen P, Macaulay V, Moral P, Olckers A (2002). A back migration from Asia to sub-Saharan Africa is supported by high-resolution analysis of human Y-chromosome haplotypes. Am J Hum Genet.

[25] Hassan HY, Underhill PA, Cavalli-Sforza LL, Ibrahim ME (2008). Y-chromosome variation among Sudanese: restricted gene flow, concordance with language, geography, and history. Am J Phys Anthropol.

[26] Scheinfeldt LB, Soi S, Tishkoff SA (2010). Colloquium paper: working toward a synthesis of archaeological, linguistic, and genetic data for inferring African population history. Proc Natl Acad Sci USA.

[27] Ogilvy SM (1981). Food habits of the Dinka in the Jonglei area of Sudan—a preliminary study. J hum nutr..

[28] Bayoumi RA, Flatz SD, Kuhnau W, Flatz G (1982). Beja and Nilotes: nomadic pastoralist groups in the Sudan with opposite distributions of the adult lactase phenotypes. Am J Phys Anthropol.

[29] Grab W, Charbonnier L. The impact of religion on social cohesion, social capital formation and social development in different cultural contexts: entering the field in international and interdisciplinary perspectives. Studien zu Religion und Kultur/Studies of Religion and Culture. Germany: LIT Verlag; 2014.

[30] Harcourt AH (2012). Human biogeography.

[31] Abdelgadir WS, Hamed SH, Moller PL, Jakobsen M (2001). Characterisation of the dominant microbiota of Sudanese fermented milk Rob. Int Dairy J..

[32] Abdelgadir W, Nielsen DS, Hamad S, Jakobsen M (2008). A traditional Sudanese fermented camel’s milk product, Gariss, as a habitat of *Streptococcus infantarius**subsp. infantarius*. Int J Food Microbiol.

